# Increased microRNA-155 expression in the serum and peripheral monocytes in chronic HCV infection

**DOI:** 10.1186/1479-5876-10-151

**Published:** 2012-07-30

**Authors:** Shashi Bala, Yaphet Tilahun, Odette Taha, Hawau Alao, Karen Kodys, Donna Catalano, Gyongyi Szabo

**Affiliations:** 1 Department of Medicine, University of Massachusetts Medical School, 364 Plantation Street, Worcester, MA 01605, USA

**Keywords:** Biomarker, MiR-122, MiR-155, MiR-125b, ALT, Inflammation

## Abstract

**Background:**

Hepatitis C Virus (HCV), a single stranded RNA virus, affects millions of people worldwide and leads to chronic infection characterized by chronic inflammation in the liver and in peripheral immune cells. Chronic liver inflammation leads to progressive liver damage. MicroRNAs (miRNA) regulate inflammation (miR-155, -146a and -125b) as well as hepatocyte function (miR-122).

**Methods:**

Here we hypothesized that microRNAs are dysregulated in chronic HCV infection. We examined miRNAs in the circulation and in peripheral monocytes of patients with chronic HCV infection to evaluate if specific miRNA expression correlated with HCV infection.

**Results:**

We found that monocytes from chronic HCV infected treatment-naïve (cHCV) but not treatment responder patients showed increased expression of miR-155, a positive regulator of TNFα, and had increased TNFα production compared to monocytes of normal controls. After LPS stimulation, miR-155 levels were higher in monocytes from cHCV patients compared to controls. MiR-125b, which has negative regulatory effects on inflammation, was decreased in cHCV monocytes compared to controls. Stimulation of normal monocytes with TLR4 and TLR8 ligands or HCV core, NS3 and NS5 recombinant proteins induced a robust increase in both miR-155 expression and TNFα production identifying potential mechanisms for in vivo induction of miR-155. Furthermore, we found increased serum miR-155 levels in HCV patients compared to controls. Serum miR-125b and miR-146a levels were also increased in HCV patients. Serum levels of miR-122 were elevated in cHCV patients and correlated with increased ALT and AST levels and serum miR-155 levels.

**Conclusion:**

In conclusion, our novel data demonstrate that miR-155, a positive regulator of inflammation, is upregulated both in monocytes and in the serum of patients with chronic HCV infection. Our study suggests that HCV core, NS3, and NS5 proteins or TLR4 and TLR8 ligands can mediate increased miR-155 and TNFα production in chronic HCV infection. The positive correlation between serum miR-155 and miR-122 increase in cHCV may be an indicator of inflammation-induced hepatocyte damage.

## Background

Hepatitis C virus infection affects over 170 million people worldwide, and the majority of those have chronic hepatitis that can lead to progressive liver disease, cirrhosis and hepatocellular cancer. Elimination of the HCV virus by the immune system is a complex process and alterations in both innate and adaptive immune responses contribute to chronic HCV infection [1]. Chronic HCV infection is characterized by the presence and activation of inflammatory cells in the liver, and the persistent inflammation contributes to liver fibrosis and liver damage [2,3]. In addition to the local inflammation in the liver, a low-grade systemic inflammation was noted in several studies, specifically, increased pro-inflammatory cytokine levels and activation of blood monocytes was found in individuals with chronic HCV infection [4,5]. Our previous studies suggested that monocytes from cHCV patients produce increased levels of TNFα, and this was related to a loss of toll-like receptor tolerance that is a mechanism in healthy cells to prevent pro-inflammatory over-activation [6]. Other studies also showed a correlation between HCV virus levels, inflammation and liver injury in chronic HCV infection [7,8].

Innate immune responses provide the first line of defense to pathogens. Innate immune responses can be mediated by pathogen-derived signals such as lipopolysaccharide (LPS) from Gram-negative bacteria that bind to toll like receptor 4 (TLR4), or HCV core (viral core protein) and NS3 (non-structural viral protein) proteins from Hepatitis C virus (HCV) that can activate cells via TLR2 [9,10]. A recent study demonstrated that NS5 (non-structural viral protein) also induces TNFα in human Kupffer cells [11]. Moreover, viral envelope protein E2 has been shown to interact with CD81 and enhances the expression of activation-induced cytidine deaminase and TNFα production in B cells [12]. The engagement of TLRs leads to the activation and expression of many genes that are directed to clear the pathogen. HCV, a single-stranded RNA virus, also interacts with TLR7/8, which recognizes small pieces of non-self nucleotides [13]. With the activation of many of the TLRs, the transcription factor NFκB is activated and subsequent inflammatory genes are transcribed. With the growing evidence for the roles of microRNAs (miRNAs) as posttranscriptional regulators of inflammation, we hypothesized that miRNAs involved in inflammation in monocytes may be dysregulated in HCV patients, thereby contributing to inflammatory cell activation.

The miRNAs relevant to monocyte inflammatory activation are miR-155, miR-146a, and miR-125b as they regulate inflammation at multiple levels [14]. MicroRNA-155 is a positive regulator of TNFα, which is a key cytokine of inflammation and regulated at the transcriptional level by the transcription factor, NF-κB. Previous studies have found elevated TNFα levels in HCV patients, thus we aimed to investigate the role of miRNAs that are known to regulate TNFα. Other miRNAs that are involved in the regulation of TNFα directly or indirectly include miR-125b and miR-146a, which are the negative regulators of TLR4/LPS signaling [14,15].

In this study, we report increased expression of miR-155 in monocytes of patients with chronic HCV infection that is no longer present in individuals who underwent successful antiviral therapy. Our results demonstrate that miR-155 is also increased in the serum of cHCV patients along with miR-122, a microRNA abundant in hepatocytes. Our studies also identify possible mechanisms for in vivo miR-155 activation in chronic HCV infection.

## Methods

### Monocyte isolation and stimulation

Monocytes were isolated from normal healthy controls and HCV infected patients using Ficoll-Paque density gradient centrifugation as described [16]. The patient information is given in detail in Table 1. Written consents were obtained from the blood donors enrolled in the study. The protocol for collecting blood is approved by the "Institutional Review Board" (IRB), called the "Committee for the Protection of Human Subjects in Research" at UMMS. LPS and CL075 were purchased from Sigma-Aldrich and Invivogen respectively. HCV NS3, NS5 and core recombinant proteins were purchased from BioDesign (USA). The experiment was set up in duplicates from the monocytes of healthy individuals. The LPS contamination of recombinant proteins was <0.01 EU/ml, detected by *Limulus* amebocyte assay as described earlier [10]. HCV E2 recombinant protein was obtained from MassBiologics (Boston, MA, USA) and contained undetectable endotoxin levels. 

**Table 1:** Clinical parameters of HCV Patients (peripheral blood monocytes isolation)

**Parameters**	**HCV patients (Average** ± **SEM)**
Gender: Responders: Male/female Naïve: Male/female	2/3 10/2
Age: Responders: Naïve:	50.50± 8.17 42.62± 4.7
AST (U/l) Naïve:	44.87± 5.2
ALT (U/l) Naïve:	81.85± 15.63
Viral load (Iu/ml): Naïve:	6X10^4^ - 9.4X10^5^ IU/ml

### RNA isolation and PCR analyses

Total RNA was isolated with the miRNeasy kit (Qiagen). For mRNA analysis, RNA was transcribed into cDNA using Promega’s reverse transcription kit (Promega) and quantitative analyses of genes were performed using gene-specific primers on a Bio-Rad iCycler real time machine. Primer sequences were: TNFα, forward 5'-ATCTTCTCGAACCCCGAGTGA-3', reverse 5'-CGGTTCAGCCACTGGAGCT-3', TLR4, forward 5'- AGGCCGAAAGGTGATTGTTG -3', reverse 5'-CTGAGCAGGGTCTTCTCCAC-3' and TLR8, forward 5'-TGTGATGGTGGTGCTTCAAT-3', reverse 5'-ATGCCCCAGAGGCTATTTCT-3'. The data was normalized to 18 S and fold change was calculated using delta-delta Ct method.

### miRNA analyses

TaqMan miRNA Assays (Applied Biosystems) were employed for the detection of miRNAs as described [17]. In monocytes, RNU48 was used to normalize the Ct values between the samples. Serum samples were collected into serum separator tubes (BD Biosciences), centrifuged at 2000 rpm, aliquoted and stored at -80°C. 100ul of serum from healthy controls and patient samples was used for total RNA isolation with miRNeasy kit (Qiagen). Synthetic C. elegans (cel)-miR-39 was spiked during the total RNA isolation process and used to normalize the real time PCR data. All PCR reactions were performed in duplicates, and miRNA data from serum samples was repeated twice. The patient information is given in Table 2. 

**Table 2:** Clinical parameters of HCV Patients used for serum miRNA isolation

**Parameters**	**HCV patients (Average** ± **SEM)**
Gender: Male/female	27/11
Age	54.07 ± 1.92
AST (U/l)	61.53 ± 7.31
ALT (U/l)	99.03 ± 19.91
Viral load (Iu/ml)	5X10^4^ – 9.3X10^6^
Genotype	1a, 1b, 2b, 3a
Category	HCV naïve: 19 HCV: 15 HCV non responders: 3 Hep B + C: 1

### Statistical analysis

The non-parametric Mann–Whitney test or two-tailed *t*-test was employed for statistical analysis and p value less than 0.05 was considered significant.

## Results

### MiR-155 expression is increased in monocytes of treatment-naïve patients with chronic HCV infection

**Figure 1:**
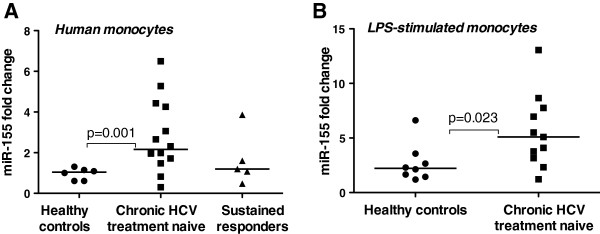
**Induction of miR-155 in peripheral monocytes of chronic HCV treatment naïve patients.A**. Total RNA was isolated from the monocytes of healthy volunteers (n = 6), chronic HCV treatment naïve patients (n = 12) and sustained responders (n = 5) with miRNeasy kit (Qiagen). **B**. Monocytes of healthy controls and chronic HCV treatment naïve patients were stimulated with 100 ng/ml LPS for 6-8 h. TaqMan miRNA assay (Applied Biosystems) was used for detection of miR-155. RNU48 was used as a normalization control. The non-parametric Mann–Whitney test was employed for statistical analysis.

Previous studies demonstrated that increased monocyte TNFα production in chronic HCV infection is causally linked to the presence of the HCV virus, circulating HCV core protein, and LPS [6,18]. Here we investigated patients with chronic HCV infection (cHCV) who were treatment-naïve and had detectable virus, sustained virological responder patients who have successfully cleared HCV infection after receiving standard therapy (HCV-responders), and healthy individuals as controls. We found that circulating monocytes of cHCV patients had significantly higher levels of miR-155 compared to sustained responders or normal controls (Figure 1A). In vitro LPS stimulation revealed that monocytes from cHCV patients had significantly higher LPS-induced miR-155 levels compared to normal control monocytes (Figure 1B). 

**Figure 2:**
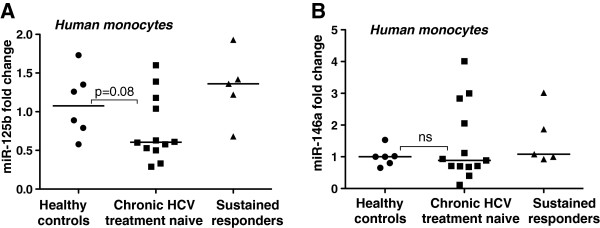
**Decreased miR-125b expression in peripheral monocytes of chronic HCV treatment naïve patients.A**-**B**. Total RNA was isolated from monocytes of healthy volunteers (n = 6), chronic HCV treatment naïve patients (n = 12) and sustained responders (n = 5) with miRNeasy kit (Qiagen). MiR-125b and miR-146a levels were detected by TaqMan miRNA assay (Applied Biosystems), and RNU48 was used as a normalization control. The non-parametric Mann–Whitney test was employed for statistical analysis.

While miR-155 increases TNFα production, miR-125b and miR-146a have negative regulatory effects on TNFα [15,19]. We found reduced miR-125b and no change in miR-146a in monocytes of patients with cHCV compared to HCV-responders and normal controls (Figure 2A and B). 

### Increased TNFα and TLR8 expression in monocytes of HCV infected patients

**Figure 3:**
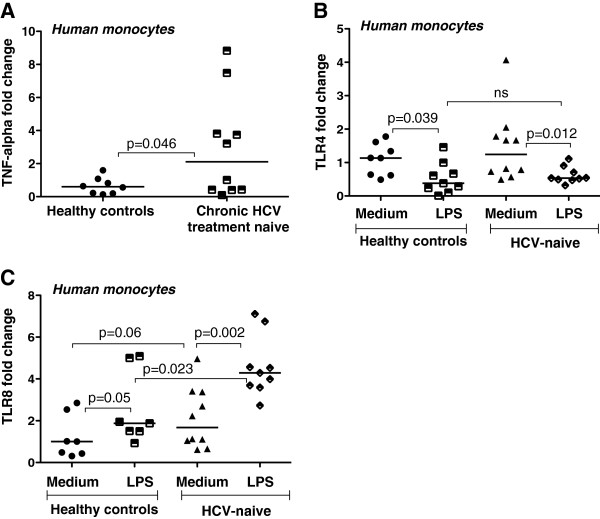
**Increased TNFα and TLR8 expression in peripheral monocytes of chronic HCV treatment naïve patients.A**-**C**. Total RNA was isolated from monocytes of healthy volunteers treated or not (n = 6-8), and from chronic HCV treatment naïve patients stimulated or not (n = 9-10) with 100 ng/ml LPS for 6-8 h. Real time PCR was carried out using gene-specific primers for TNFα (**A**), TLR4 (**B**) and TLR8 (**C**) expression. 18 S was used as a normalization control and the non-parametric Mann–Whitney test was employed for statistical analysis.

In a previous study, we reported increased TNFα production by monocytes from patients with cHCV [6]. Here, we also found increased TNFα mRNA expression in monocytes of cHCV patients (Figure 3A). Because increased TNFα production could be induced by ligands for TLR4 (LPS, NS5), TLR2 (HCV core and NS3 proteins) or TLR8 (single-stranded RNA), we evaluated the expression of these TLRs in monocytes. We found no difference in baseline TLR4 expression between monocytes from cHCV infected patients and normal controls, and TLR4 mRNA was decreased in both groups after LPS stimulation (Figure 3B). In contrast, TLR8 mRNA levels were significantly higher in monocytes from cHCV-infected patients and showed upregulation after an in vitro LPS challenge (Figure 3C). There were no significant changes in TLR1, TLR2 and TLR6 levels in monocytes between cHCV and normal controls with or without LPS stimulation (data not shown). 

### HCV core, NS3 and NS5 proteins and TLR4 and TLR8 ligands increase miR-155 levels in human monocytes

**Figure 4:**
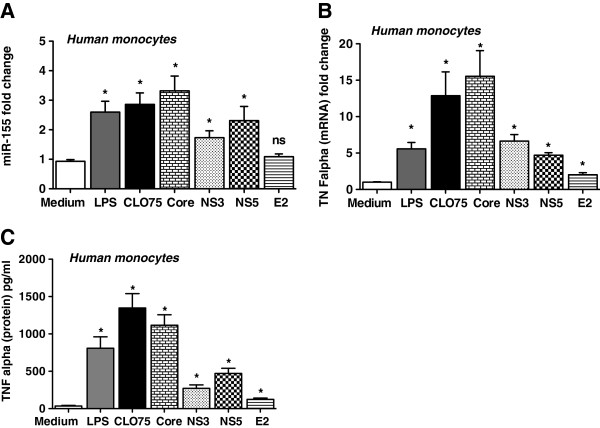
**Viral NS3, NS5 and core proteins and TLR4 and TLR8 ligands induce miR-155 and TNFα in monocytes of healthy controls.A**-**B**. Monocytes of healthy controls (n = 5) were stimulated or not with 100 ng/ml LPS (TLR4 ligand), 5ug/ml CLO75 (TLR8 ligand), 5ug/ml viral core protein, NS3 and NS5 proteins (non-structural viral proteins), and E2 (viral envelop protein) for 6-8 h. The experiment was performed in duplicates. A. Total RNA was isolated and subjected to TaqMan miRNA assay for miR-155. B&C. TNFα mRNA expression was examined by real time PCR (**B**) and protein levels were determined by ELISA in cell-free supernatants (**C**). Two-tailed *t*-test was used for statistical analysis.

MicroRNA-155 has recently been identified as a positive regulator of TNFα production [19]. Because increased expression of TNFα in the liver and in blood monocytes was previously found in cHCV and TLR ligands, and HCV proteins were shown to induce TNFα in monocytes, we tested the effect of these TLR ligands on miR-155 induction [6,10,20]. Here we found increased miR-155 levels in normal monocytes after stimulation with the TLR4 ligand, LPS (Figure 4A). HCV is a single stranded RNA virus that can be recognized by TLR7 and/or TLR8 of which TLR8 is expressed in monocytes [10,16]. We found that the TLR8 ligand, CLO75, increased miR-155 levels in monocytes compared to unstimulated cells (Figure 4A). Multiple mechanisms have been suggested for increased TNFα production in monocytes of patients with chronic HCV infection including circulating HCV core protein [6,9,21]. Our results show that stimulation with recombinant HCV core or NS3 or NS5 proteins significantly increased monocyte miR-155 expression in normal human monocytes (Figure 4A). In contrast, E2 (viral envelop protein, E2) failed to induce miR-155 expression in monocytes (Figure 4A). Parallel to miR-155 induction, LPS, CLO75, HCV core, NS3 and NS5 all induced significant increases in TNFα mRNA (Figure 4B) and protein levels (Figure 4C). Furthermore, we found that E2 protein also resulted in a moderate but significant increase in TNFα, both at mRNA and protein levels (Figure 4B & C). 

### Increased miR-155 in the serum of patients with chronic HCV infection

**Figure 5:**
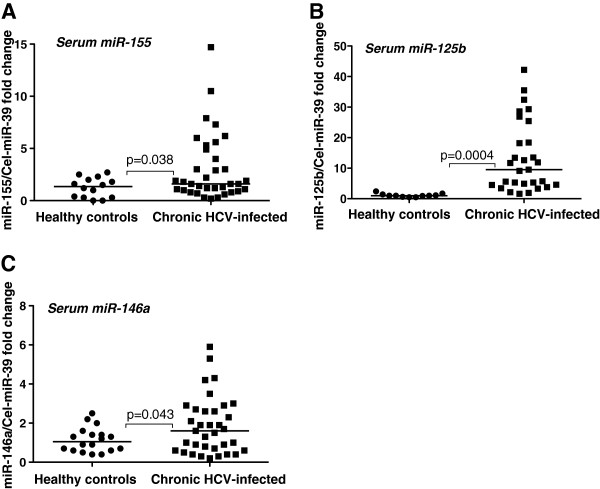
**Increased serum miR-155, miR-125b and miR-146a levels in chronic HCV-infected patients.A**-**C**. 100ul of serum was used for total RNA isolation from both healthy controls (n = 12-18) and chronic HCV patients (n = 30-36) with miRNeasy kit (Qiagen). TaqMan miRNA assays (Applied Biosystems) were used to detect miRNA levels and C.elegans (Cel)-miR-39 was used to normalize the ct values. The non-parametric Mann–Whitney test was used for statistical analysis.

Increasing evidence suggests that circulating micro-RNAs may serve as biomarkers in disease conditions [22]. Here we tested serum levels of miR-155 and found a statistically significant increase in serum miR-155 levels in patients with chronic HCV infection (cHCV) compared to normal controls (Figure 5A). Serum levels of miR-125b and miR-146a were also increased in cHCV patients compared to normal controls (Figure 5B and C). 

### Increased levels of miR-122 in the serum of patients with chronic HCV infection

**Figure 6:**
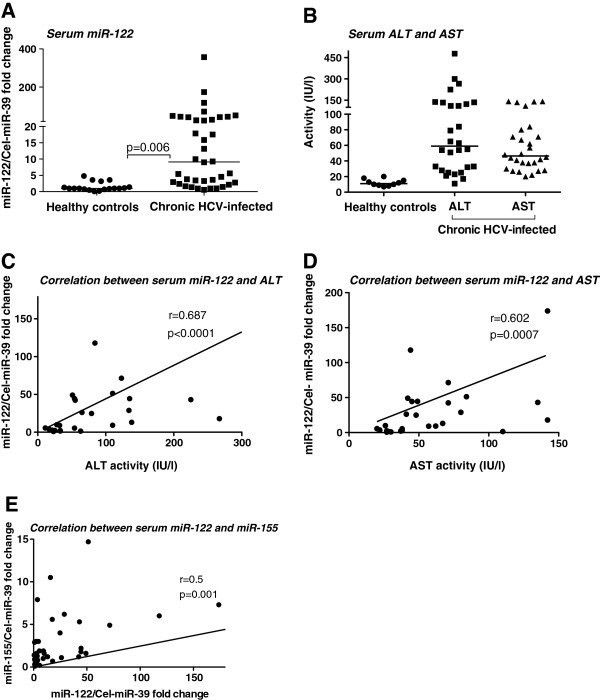
**Increased circulating miR-122 levels correlate with serum ALT, AST and miR-155 levels in chronic HCV-infected patients.A**. 100ul of serum was used for total RNA isolation from both healthy controls (n = 12-13) and chronic HCV patients (n = 33) with miRNeasy kit (Qiagen). Serum miR-122 levels were detected with TaqMan miRNA assay as described in the methods. The non-parametric Mann–Whitney test was used for statistical analysis. **B**. Serum ALT or AST levels of healthy and chronic HCV-infected individuals. **C**-**E**. Correlation between serum miR-122 and ALT (**C**) and AST (**D**) and serum miR-155 (**E**) was determined with the Spearman method.

Elevated serum or plasma miR-122 has been found in correlation with liver injury in recent studies [23-26]. We found that patients with chronic HCV infection had significantly increased serum miR-122 levels compared to controls (Figure 6A). In the same patients, serum levels of ALT and AST were also elevated (Figure 6B) and this finding was consistent with the presence of chronic HCV infection. There was a strong correlation between serum ALT/AST and serum miR-122 increases (Figure 6C&D). Importantly, a correlation between serum miR-155 and miR-122 levels was found in cHCV patients (Figure 6E). These data suggest that circulating micro-RNAs may reflect disease activity in chronic HCV infection. 

## Discussion

Chronic hepatitis from HCV infection is characterized by ongoing immune activation and a low-grade inflammation in the liver and in the periphery that accompanies the liver injury [13,27]. While numerous studies reported the presence of inflammatory cascade activation, the mechanisms that trigger and sustain the increased TNFα and inflammatory cell activation remain elusive [2,27,28]. Micro-RNAs have emerged as key regulators of inflammation of which miR-155 has a unique positive regulatory effect on TNFα production [19,29]. In this report, we tested the hypothesis that micro-RNAs are dysregulated in chronic HCV infection. We found that treatment-naïve patients with chronic HCV infection have increased expression of miR-155 in their circulating monocytes that are the major sources of TNFα production. Importantly, miR-155 levels were not increased in monocytes from patients who have successfully cleared HCV infection after therapy, suggesting a possible correlation between increased miR-155 and HCV viral presence and/or replication. We also found that cHCV monocytes have increased mRNA expression of TLR8, a receptor for single-stranded RNA and showed that in normal monocytes not only TLR4 and TLR8 ligands but HCV core, NS3 and NS5 proteins induced miR-155 upregulation. MiR-155 was elevated both in the monocytes and in the serum compartment in chronic HCV infection, and this occurred in the presence of increased serum miR-122. Together, our results suggest that miR-155 upregulation in HCV infection in monocytes may contribute to the increased pro-inflammatory state and raises the possibility that circulating miRNAs could serve as disease markers.

Increased miR-155 levels in circulating monocytes of HCV patients may have several implications for disease development and progression. First, miR-155 over-expression in monocytes and macrophages has been shown to sensitize to stimulation with LPS [17,19]. Indeed, we not only found increased baseline miRNA-155 expression but also a significant upregulation of miR-155 after in vitro LPS challenge in cHCV infection. Previous studies from our and other laboratories demonstrated that monocytes from chronic HCV infected patients have increased baseline TNFα production as well as hyper-responsiveness to ex vivo stimulation with LPS, suggesting that increased miR-155 levels may contribute to the over-activation of the pro-inflammatory phenotype of monocytes. This notion is supported by reports where knock-down of miR-155 or miR-155-deficient mice showed attenuated TNFα production to LPS stimulation [17,30,31]. Intriguingly, various studies have demonstrated TNFα as a positive regulator of miR-155, indicating a positive feed back regulation [32]. Based on our results, it is tempting to speculate that increased miR-155 sensitizes monocytes to a TLR-induced trigger for TNFα production in active HCV infection. In parallel to our work, a recent study showed that expression of BIC gene, a precursor of mature miR-155, was increased in PBMCs of HCV-infected patients [33]. They further demonstrated that the highest expression of BIC was found in patients harboring HCV RNA in serum and PBMCs whereas lowest BIC expression was observed in patients that eliminated HCV RNA from both serum and PBMCs after anti-viral treatment, suggesting a correlation between HCV RNA and precursor levels of miR-155 [33]. Moreover, a correlation between miR-155 expression and HCV replication has been demonstrated in PBMCs of chronic hepatitis C patients [34]. Our findings further indicate an increase of mature miR-155 in monocytes and serum of cHCV patients. Although we found no significant changes in miR-146a levels in monocytes of cHCV patients, some of patients who had higher miR-146a levels also showed higher levels of miR-155 and miR-125b. A large-scale clinical study might answer if the variability between miRNAs correlates with HCV viral load or progression of HCV infection.

Our observations also suggest that HCV-related TLR ligands provide the possible mechanisms for in vivo induction of miR-155 and monocyte activation in cHCV patients [13]. For example, high levels of HCV core protein as well as LPS are present in patients with cHCV [6,35]. Our data show that both HCV core protein and LPS induce miR-155 in normal monocytes. It has been shown that HCV core protein can activate monocytes and DCs via TLR2 and the complement C1q receptor [9,18]. In addition, HCV NS3 protein that activates TLR2, also increased monocyte miR-155 in our hands. A recent study reported the induction of TNFα in response to NS5 (non-structural viral protein) treatment in human Kupffer cells [11]. In agreement with this study, we found induction of miR-155 and TNFα in monocytes treated with NS5. NS5 has been shown to activate the TLR4 pathway [36]. Previously, it has been shown that viral E2 protein interacts with CD81 and increases the expression of activation-induced cytidine deaminase and TNFα production in B cells [12]. We also found a moderate increase of TNFα in monocytes treated with E2 protein without any change in miR-155 levels. Our data suggest that viral E2 protein can induce TNFα independent of miR-155, however, further studies are needed to confirm these findings. Finally, we found that TLR8 ligand activation can increase miR-155 and TNFα production and it is feasible that HCV, as a single-stranded RNA, induces miR-155 in monocytes via TLR8. Together our data demonstrate that multiple, HCV-associated TLR ligands are likely to be involved in miR-155 induction in cHCV infection.

We not only found increased miR-155 and miR-122 levels in the serum of cHCV patients but levels of miR-125b and miR-146a were also increased. Increased circulating miR-125b levels have been found in various cancers and recently, it has been reported that increased circulating miR-125b levels were associated with chemotherapeutic resistance in breast cancer [37]. The significance of increased miR-125b in HCV patients awaits further investigation. Circulating miR-146a levels were shown to be increased in patients with type 2 diabetes [38]. Interestingly, HCV is known to induce type 2 diabetes [39], and it is tempting to speculate that increased miR-146a in the circulation might predispose the individuals to HCV infection or vice versa.

Although, there are many studies depicting the increased circulating miRNAs in various etiologies, the source of circulating miRNAs is not as clear. The liver is a complex organ where various cell types reside and interact in close vicinity. In our study, there could be multiple factors that contribute to induction of miRNAs (miR-122, miR-155, miR-146a and miR-125b) in the circulation during HCV infection. The possibility that these miRNAs are either released from immune cells in a non-specific manner or are released from different cell types in a cell-specific manner in response to HCV infection cannot be ruled out. It is most likely that miR-122 is released from damaged hepatocytes. In agreement with this, we found increased levels of miR-122 in the supernatant of JFH-1 or J6/JFH-1 infected HuH7.5 cells (data not shown). Recent studies suggest that miR-155 is not only limited to immune cells (dendritic cells, Kuffper cells, monocytes, NK cells, T cells), but also prevalent in non-immune cells (hepatocytes, endothelial cells). We have recently reported that chronic alcohol feeding increases miR-155 not only in Kuffper cells but also in hepatocytes [40], and it has been shown that alcoholic hepatitis patients have a higher prevalence of HCV infection [41]. Our preliminary results suggest a moderate increase of miR-155 in the supernatant of JFH-1 or J6/JFH-1 infected cells compared to uninfected HuH7.5 cells (data not shown). On the basis of these findings, it is most likely that both immune cells and hepatocytes contribute to increase of miR-155 in the circulation in HCV infection. Further studies are warranted to investigate the cellular source of circulating miRNAs.

Growing evidences suggest that circulating miRNAs may be affected by age and body weight and it was reported that levels of circulating miRNA-155 inversely correlated with age in patients with coronary artery disease [42]. Whether these factors affect the outcome of circulating miRNAs in HCV patients needs to be investigated. Finally, we found increased miR-122 levels in the serum of chronic HCV infected patients, which strongly correlated with liver damage markers such as ALT and AST. The correlation of serum miR-122 was more robust with ALT than AST. Our results support the previous findings where a strong correlation between serum miR-122 and ALT/AST increases has been reported [43,44]. Interestingly, our data indicates a positive correlation between serum miR-155, an inflammation-related miRNA, and miR-122, a hepatocyte-specific micro-RNA. We speculate that this could be related to inflammation-induced hepatocyte damage that occurs in chronic HCV infection. In our patient cohort, there was no significant correlation between ALT values and serum miR-155 expression. Further investigation of serum miRNAs and their role as diagnostic biomarkers or biological mediators deserves attention.

## Conclusions

Our study demonstrates that monocytes have increased miR-155 expression and increased TNFα production in patients with chronic HCV infection. The observation that miR-155 is induced in monocytes by TLR4 and TLR8 stimulation as well as by HCV core, NS3 and NS5 proteins suggests potential mechanisms for in vivo miR-155 induction in chronic HCV infection. Our finding of increased serum miR-155 and miR-122 and their correlation implies that microRNAs deserve investigations as potential biomarkers in disease activity.

## Competing interests

The authors declare that they have no competing interests.

## Authors’ contribution

SB conceived the idea, performed the experiments, analyzed the data and wrote the manuscript. YT carried out miRNA isolation and analyzed the PCR data from monocytes. OT performed miRNA isolation and PCR analysis from monocytes. HA performed the miRNA isolation and PCR from serum samples. KK performed the miRNA isolation and PCR experiments from monocytes and serum samples. DC carried out ELISA and monocytes isolation. GS conceived the idea, supervised the project, wrote the manuscript and obtained the funding. All the authors read and approved the final manuscript.
